# Forced expression of muscle specific kinase slows postsynaptic acetylcholine receptor loss in a mouse model of MuSK myasthenia gravis

**DOI:** 10.14814/phy2.12658

**Published:** 2015-12-23

**Authors:** Nazanin Ghazanfari, Erna L. T. B. Linsao, Sofie Trajanovska, Marco Morsch, Paul Gregorevic, Simon X. Liang, Stephen W. Reddel, William D. Phillips

**Affiliations:** ^1^ Physiology and Bosch InstituteUniversity of SydneySydneyNew South WalesAustralia; ^2^ Department of Biomedical SciencesMacquarie UniversitySydneyNew South WalesAustralia; ^3^ Baker IDI Heart and Diabetes InstituteMelbourneVictoriaAustralia; ^4^ Department of Biochemistry and Molecular BiologyCollege of Basic Medical SciencesLiaoning Medical UniversityLiaoningChina; ^5^ Department of Molecular MedicineConcord HospitalSydneyNew South WalesAustralia

**Keywords:** myasthenia, neuromuscular junction, synapse formation, tyrosine kinase

## Abstract

We investigated the influence of postsynaptic tyrosine kinase signaling in a mouse model of muscle‐specific kinase (MuSK) myasthenia gravis (MG). Mice administered repeated daily injections of IgG from MuSK MG patients developed impaired neuromuscular transmission due to progressive loss of acetylcholine receptor (AChR) from the postsynaptic membrane of the neuromuscular junction. In this model, anti‐MuSK‐positive IgG caused a reduction in motor endplate immunolabeling for phosphorylated Src‐Y418 and AChR *β*‐subunit‐Y390 before any detectable loss of MuSK or AChR from the endplate. Adeno‐associated viral vector (rAAV) encoding MuSK fused to enhanced green fluorescent protein (MuSK‐EGFP) was injected into the tibialis anterior muscle to increase MuSK synthesis. When mice were subsequently challenged with 11 daily injections of IgG from MuSK MG patients, endplates expressing MuSK‐EGFP retained more MuSK and AChR than endplates of contralateral muscles administered empty vector. Recordings of compound muscle action potentials from myasthenic mice revealed less impairment of neuromuscular transmission in muscles that had been injected with rAAV‐MuSK‐EGFP than contralateral muscles (empty rAAV controls). In contrast to the effects of MuSK‐EGFP, forced expression of rapsyn‐EGFP provided no such protection to endplate AChR when mice were subsequently challenged with MuSK MG IgG. In summary, the immediate in vivo effect of MuSK autoantibodies was to suppress MuSK‐dependent tyrosine phosphorylation of proteins in the postsynaptic membrane, while increased MuSK synthesis protected endplates against AChR loss. These results support the hypothesis that reduced MuSK kinase signaling initiates the progressive disassembly of the postsynaptic membrane scaffold in this mouse model of MuSK MG.

## Introduction

Most cases of myasthenia gravis (MG) are caused by autoantibodies that target the nicotinic acetylcholine receptor (AChR) in the postsynaptic membrane of the neuromuscular junction (NMJ; Berrih‐Aknin et al. 2014). The major pathogenic mechanisms involve bivalent AChR autoantibodies cross‐linking adjacent AChR to cause accelerated AChR degradation (antigenic modulation) and complement‐mediated damage to the postsynaptic membrane (Baggi et al. 2012; Tüzün and Christadoss 2013; Ban and Phillips 2015). However, a proportion of MG patients instead express autoantibodies that target the extracellular domains of muscle‐specific (tyrosine) kinase (MuSK; Hoch et al. 2001; McConville et al. 2004; Reddel et al. 2014) or its co‐receptor, low‐density lipoprotein receptor‐related protein 4 (LRP4; Higuchi et al. 2011; Pevzner et al. 2012; Zhang et al. 2012; Zisimopoulou et al. 2014). The mechanisms through which MuSK autoantibodies cause the loss of postsynaptic AChR at the NMJ in vivo remain to be fully defined.

Our understanding of the pathogenesis of MuSK autoantibodies depends upon animal and cell culture experiments (Phillips et al. 2015). Rabbits, rats, and mice that were actively immunized with the extracellular domain of MuSK developed MuSK autoantibodies. They showed impairment of neuromuscular transmission associated with loss of postsynaptic AChR and other changes to the NMJ structure (Jha et al. 2006; Shigemoto et al. 2006; Xu et al. 2006; Richman et al. 2011; Chroni and Punga 2012; Viegas et al. 2012; Patel et al. 2014; Ulusoy et al. 2014). Passive transfer of IgG from MuSK MG patients into mice also caused loss of postsynaptic AChR and synaptic impairments (Cole et al. 2008; Viegas et al. 2012). Most of the autoantibodies from MuSK MG patients are of the IgG4 subtype (McConville et al. 2004), and it has been demonstrated that administering the IgG4 fraction from MuSK MG plasma to mice was sufficient to cause myasthenia gravis (Klooster et al. 2012). However, antibodies of the IgG4 subtype are considered functionally monovalent and would not be expected to cross‐link MuSK or activate complement (Tao et al. 1991; Aalberse et al. 2009). These observations suggest that MuSK autoantibodies drive the development of pathology via an alternative mechanism. A proteoglycan called neural agrin (secreted by the motor nerve) can activate MuSK by binding to its co‐receptor, LRP4 (Kim et al. 2008; Zhang et al. 2008). Upon agrin‐induced activation of MuSK, nonreceptor tyrosine kinases including Abl and Src become phosphorylated, leading to tyrosine phosphorylation of the AChR *β*‐subunit (Mohamed et al. 2001; Smith et al. 2001; Mittaud et al. 2004). Phosphorylation of the AChR recruits the cytoplasmic protein rapsyn (Borges et al. 2008). Hypothetically, this extra rapsyn might stabilize endplate AChR by: (1) cross‐linking adjacent AChR (Zubera and Unwin 2013); (2) anchoring AChR to the cytoskeleton (Lo et al. 1980; Phillips et al. 1993); (3) recruiting protein kinase A, which fosters the local recycling of AChR (Choi et al. 2012); and (4) inhibiting calpain and caspase‐3, which may protect the AChR cluster from calcium‐mediated disassembly mechanisms (Chen et al. 2007; Wang et al. 2014). In cell culture experiments, MuSK autoantibodies disrupted the binding of LRP4 to MuSK, thereby preventing the activation of MuSK by neural agrin (Huijbers et al. 2013; Koneczny et al. 2013). These in vitro findings raise the hypothesis that MuSK autoantibodies disrupt maintenance of the adult NMJ in vivo simply by suppressing MuSK signaling function within the postsynaptic membrane. Here, we examine the early pathophysiological changes at the NMJ and the influence of MuSK expression on disease progression in a mouse model of MuSK MG.

The passive transfer mouse model has been used to examine the effects of MuSK autoantibodies on the NMJ in vivo. Mice administered daily injections of IgG from MuSK MG patients displayed progressive declines in endplate AChR density and endplate potential amplitude, culminating in failure of neuromuscular transmission after 12–15 days (Morsch et al. 2012, 2013). Src kinase (Y418) and the *β*‐subunit of the AChR (*β*‐subunit Y390) are key targets of the tyrosine kinase cascade initiated following MuSK activation (Mittaud et al. 2004). After 14 daily injections of IgG from MuSK MG patients, motor endplate immunolabeling for the phosphorylated Src‐Y418 (pSrc) and AChR *β*‐subunit‐Y390 (pAChR) were both diminished, consistent with the idea that anti‐MuSK IgG suppresses MuSK kinase activity within the postsynaptic membrane (Ghazanfari et al. 2014). However, at this late stage of the disease process, postsynaptic immunofluorescence for MuSK and AChR were also diminished (Cole et al. 2010; Ghazanfari et al. 2014). Developmental studies have suggested that structural interactions of MuSK with other proteins might play a role in organizing the specialized postsynaptic membrane (Apel et al. 1997; Zhou et al. 1999; Bromann et al. 2004). Thus, it is possible that the loss of endplate AChR results either from reduced MuSK kinase activity or physical displacement of MuSK from the postsynaptic membrane. Here, we show that a single injection of MuSK MG patient IgG caused reductions in endplate levels of phosphorylated Src and AChR within 24 h of a single injection of MuSK MG patient IgG. Repeated daily injections of MuSK MG patient IgG caused progressive loss of AChR from the endplate. However, forced expression of recombinant MuSK provided protection against this AChR loss, despite the continually circulating activation‐blocking autoantibodies.

## Methods

### Ethical approval

All mouse experiments were conducted with the approval of the University of Sydney Animal Ethics Committee in compliance with the NSW Animal Research Act 1985 and the Australian Code of Practice for the Care and Use of Animals for Scientific Purposes 8th Edition NHMRC 2013. Six‐week‐old female C57BL/6J mice (approximately 19 g) were obtained from the Animal Resources Centre, Western Australia. Mice were fed ad libitum and had continuous access to water. A total of 35 mice were used in the study and the group size for each experiment was 3, unless otherwise stated. The authors understand the ethical principles under which this journal operates and confirm that the work described in this paper conforms to the journal animal ethics checklist. Concerning the use of human IgG, informed patient consent was obtained in accordance with the Declaration of Helsinki. The project was approved by the Human Research Ethics Committee of Sydney South West Area Health Service.

### Adeno‐associated viral vector expression constructs and injections

Full‐length murine MuSK coding sequence fused in‐frame at its 3′ (cytoplasmic) end to enhanced green fluorescent protein (MuSK‐EGFP) was described previously (Cole et al. 2008). The 3.4 kb MuSK‐EGFP open reading frame was blunt cloned into the plasmid backbone, pAAV‐CMV‐MCS‐SV40pA prior to packaging into recombinant adeno‐associated virus serotype 6 capsid (rAAV) as described (Blankinship et al. 2004). The 1.9 kb rapsyn‐EGFP open reading frame (Gervásio and Phillips 2005) was similarly blunt‐end cloned into the above vector plasmid and packaged into rAAV.

For anesthesia, mice were first placed in an induction chamber supplied with a continual source of 4% isoflurane in oxygen, until rendered unconscious. The mouse was then transferred to a procedure space and a facemask was fitted, to maintain a plane of anesthesia at which the foot withdrawal reflex remained fully suppressed (1.5–2% isoflurane). The skin of the lower hind limbs was shaved and cleaned with 70% ethanol prior to a surgical incision to expose the lateral surface of the tibialis anterior (TA) muscle. A Hamilton syringe with a 26‐gauge needle was used to inject 10 *μ*L of 0.9% sterile NaCl solution containing rAAV‐MuSK‐EGFP or rAAV‐rapsyn‐EGFP into the belly of the right TA muscle. The dose was 2 × 10^9^ viral genomes (vg), unless otherwise specified. In a pilot experiment, this dose yielded MuSK‐EGFP fluorescence at the majority of endplates (Figure S1). The left TA muscle (contralateral control) received the same dose of empty rAAV vector (no gene inserted). The incisions were suture closed and the mice were administered injections of buprenorphine (0.03 mg/kg subcutaneously; Reckitt Benckiser, Australia) immediately, and again 24 h postsurgery for analgesia.

### Passive transfer of MuSK MG IgG

**Figure 1:**
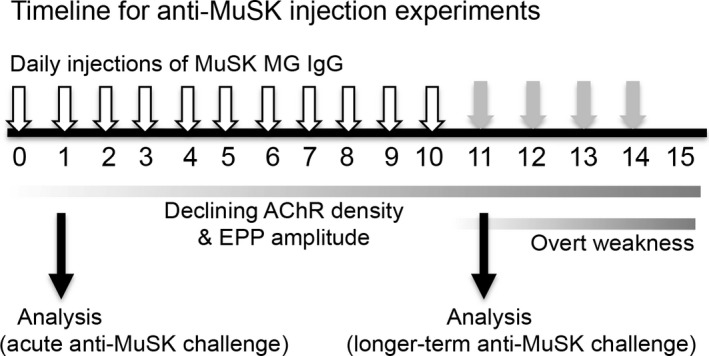
Timeline for the IgG passive transfer experiments. Mice received daily i.p. injections of MuSK MG patient IgG or non‐myasthenic control IgG (open arrows). This results in progressive declines in endplate AChR labeling and endplate potential amplitude. Weight loss and whole body weakness develops by days 12–15 (Cole et al. 2008; Morsch et al. 2012). In this study, the acute (immediate) impact of MuSK MG IgG upon the motor endplate was assessed 24 h after the first IgG injection. The longer term effects were assessed (in a second set of mice) at day 11. Recombinant adeno‐associated viral vector encoding MuSK‐EGFP or rapsyn‐EGFP was injected unilaterally into the right TA muscle several weeks prior to the first IgG injection. The contralateral (control) muscle was injected with empty rAAV vector.

The passive transfer model of MuSK MG has been detailed previously (Cole et al. 2008; Morsch et al. 2012; Phillips et al. 2015). Figure 1 illustrates the experimental design for this study. Mice received daily i.p. injections of IgG derived from therapeutic plasma exchange of anti‐MuSK‐positive MG patients (described below). To assess the immediate impact of the autoantibodies, a subset of mice was killed 24 h after a single i.p. injection of MuSK MG patient IgG. To assess the longer term effects of anti‐MuSK, another subset of mice were killed after 11 daily i.p. injections of patient IgG. Overt weakness does not develop in this model until day 13–14. To suppress any active immune response to the human proteins, the latter mice received a single i.p. injection of cyclophosphamide monohydrate (300 mg/kg; Sigma, St Louis MO; 10 mg/mL in 0.9% NaCl) 24 h after the first IgG injection (Toyka et al., 1975). On each day of the IgG injection series, mice were weighed, inspected for general health, and were graded for whole body weakness, before and after a standard exercise regime (Cole et al. 2008; Phillips et al. 2015). Mice were terminated if they either developed grade 2 weakness (mouse lying prone before exercise) or lost 15% of its original body weight. None of the mice in this study reached either of these criteria. Mice were killed with pentobarbitone (30 mg i.p.; Cenvet Australia).

The method of patient IgG purification and the batch numbering scheme was described previously (Cole et al. 2008; Ghazanfari et al. 2014). Patient 4 was at Myasthenia Gravis Foundation of America (MGFA) grade 3B when batch AM4.5 was collected. Patient 2 was at MGFA grade 4B for batch AM2. Patient 7 was at MGFA grade 3B for AM7.2. Control IgG was purified from pooled plasma arising from therapeutic venesection of hemochromatosis patients and was screened negative for both anti‐AChR and anti‐MuSK by standard clinical immunoprecipitation assays. The specific batches and amounts of IgG injected for each experiment are specified in the figure legends.

### Electromyography

Compound muscle action potentials (CMAPs) were recorded from the TA muscles of mice during repetitive nerve stimulation (Morsch et al. 2012; Plomp et al. 2015). Mice were anesthetized with isoflurane/oxygen as described above. The skin was prepared with abrasive skin prepping gel (Nuprep, D.O. Weaver & Co, Aurora). Two custom‐made single monopolar 3 mm recording electrodes were glued to the skin: one over the ventral aspect of the TA muscle and the second electrode at the ankle of the same hind limb. Electrolyte gel (VIASYS Healthcare, Madison WI) was applied directly at the electrode sites. Stimulation of the sciatic nerve was accomplished via a 4 mm incision in the sciatic notch and by placing the nerve on a custom‐made silver hook electrode (0.6 mm diameter). Animals received at least 3 trains of 10 stimuli and the recordings obtained were averaged. At the end of the CMAP recordings, the mice were killed with pentobarbitone as described above.

### Immunolabeling and confocal imaging

Muscles were dissected and fixed for 2 h in 2% paraformaldehyde/phosphate‐buffered saline (PBS) at room temperature. After fixation, muscles were washed three times with PBS over 30 min and were immersed in 20% sucrose/PBS overnight at 4°C. The following day, the muscles were frozen for sectioning as described (Tse et al. 2014). Sections were preincubated for 1 h in 2% bovine serum albumin (BSA) in PBS. To compare the relative abundance of MuSK at the endplate, 12 *μ*m transverse sections were probed overnight at 4°C with affinity purified sheep anti‐MuSK (1:100), which binds the extracellular domain of both endogenous MuSK and MuSK‐EGFP. Its binding is not blocked by preincubation with high concentrations of MuSK MG patient IgG (Cole et al. 2010). After washing in PBS, sections were incubated with Alexa647‐*α*‐bungarotoxin (1:200; Alexa647‐BGT; Molecular Probes) and affinity‐purified tetramethylrhodamine isothiocyanate (TRITC)‐conjugated donkey anti‐sheep IgG (1:250; H+L; Jackson ImmunoResearch Laboratories, Baltimore, PA) for 1 h at room temperature. Immunolabeling for phosphorylated Src‐Y418 (pSrc) and AChR *β*‐subunit‐Y390 (pAChR) were performed as described (Ghazanfari et al. 2014). Transverse cryosections of snap frozen fresh muscle (10 *μ*m) were fixed on the slide with 2% paraformaldehyde/PBS‐containing Sigma phosphatase inhibitor cocktail No. 2 (1:100, Sigma St Louis) at room temperature for 15 min. Sections were washed 3 × 10 min in PBS, incubated for 30 min in 0.3 mol/L glycine/PBS and rinsed in PBS. Slides were then incubated in methanol for 7 min and washed twice in PBS. Sections were preincubated for 1 h in blocking solution (0.2% Triton X100, 2% BSA, PBS) and incubated overnight at 4°C with either rabbit anti‐phospho‐Src‐pTyr^418^ IgG (1:300; Sigma), or rabbit polyclonal anti‐pACh‐receptor*β*1‐Tyr^390^ IgG (1:250; Santa Cruz Biotechnology) diluted in blocking solution. After washing thrice with PBS, sections were incubated for 1 h at room temperature with Cy^™^3‐conjugated Donkey Anti‐Rabbit IgG (1:250, ABACUS ALS) and Alexa‐647‐*α*‐BGT to label AChR (1:200, Invitrogen Australia). After washing thrice with PBS, coverslips were mounted with polyvinyl alcohol mounting medium containing 1,4diazabicyclo[2.2.2]octane to reduce photobleaching (DABCO, Sigma, St Louis, MO).

To permit comparison of endplate staining intensities across all sections in a given experiment, all samples were stained together on the same day and optical sections were collected in the same imaging session with fixed settings on a Zeiss LSM 510 Meta confocal microscope using a 40× 1.2 NA air objective. The confocal aperture was set to 1.0 Airy unit. For enface imaging of endplates in 20 *μ*m longitudinal sections, Z‐series were collected with optical section intervals of 1 *μ*m and a scan speed of 7. Each optical section was the average of two scans.

### Image analysis

ImageJ 1.31 v software was used to measure the relative fluorescence intensities using recently published protocols (Tse et al. 2014). A minimum of 20 endplates from each muscle were averaged. To assess the relative intensity of MuSK‐EGFP fluorescence in the postsynaptic membrane in Figure 5, the ImageJ “plot profile” tool was applied to a line transecting the endplate in a muscle fiber cross‐section. The first fluorescence peak represented the endplate while the second peak represents the relative density of MuSK‐EGFP in extrasynaptic sarcolemma (on the opposite edge of the muscle fiber). The endplate/sarcolemma peak height ratio therefore reflects the degree to which MuSK‐EGFP was concentrated at the endplate. The cytoplasmic EGFP fluorescence intensity was averaged for the cytoplasmic portion of the line profile. Differences in the dosage of rAAV injected (2.5, 7.5, 10, 15 & 20 × 10^8^ vector genomes per muscle) did not alter the endplate/sarcolemmal EGFP intensity ratio nor the endplate/cytoplasm ratio (Figure S2).

### Statistics

GraphPad Prism (GraphPad Software, CA) was used for statistical analysis. Comparison between two groups of mice was by unpaired, two‐tailed Student's *t*‐test (e.g., anti‐MuSK‐injected vs control mice) where *n* was the number of mice per treatment group. Paired *t*‐tests were used to compare MuSK‐EGFP‐expressing right TA muscles with contralateral control muscles (injected with empty rAAV vector) in the same mouse. Again, *n* was the number of mice. Significance is indicated throughout as follows: *P <* *0.05, **P <* *0.01, ***P <* *0.001.

## Results

### Immediate effects of anti‐MuSK IgG upon the postsynaptic MuSK pathway

**Figure 2:**
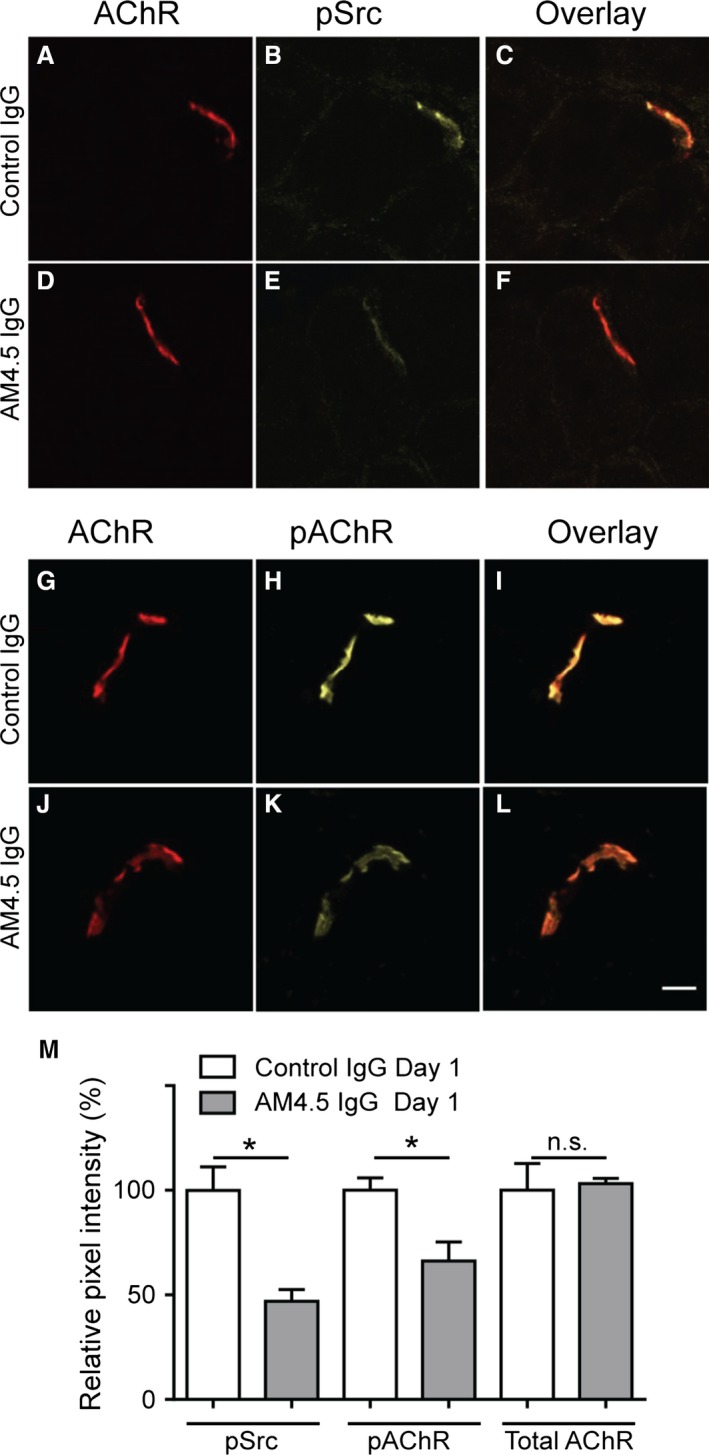
MuSK MG IgG causes a rapid reduction in endplate phosphorylation. Naive mice were challenged with a single i.p. injection of MuSK MG IgG (AM4.5; 35 mg) or non‐myasthenic control IgG (35 mg) and were killed for analysis 24 h later. (A) Transverse optical section through an endplate of a mouse injected with control IgG showing bright AChR labeling (Alexa647‐*α*‐BGT). (B) The same endplate showing bright pSrc immunofluorescence. (C) Overlay of the two fluorescence pseudo colors. (D–F) Endplate of a mouse injected with AM4.5 IgG showing bright AChR labeling, but dim pSrc immunofluorescence. (G–I) Endplate of a mouse injected with control IgG showing bright AChR labeling (Alexa647‐*α*‐BGT) and bright immunofluorescence for phosphorylated AChR (pAChR). (J–L) Endplate of a mouse injected with AM4.5 IgG showing bright AChR labeling, but dim pAChR immunofluorescence. Scale bar is 10 *μ*m. (M) Relative intensity of endplate labeling for pSrc, pAChR, and total AChR (Alexa647‐*α*‐BGT). Mice injected with AM4.5 IgG (filled bars) are compared to mice injected with control IgG (open bars). Bars represent the mean ± SEM for *n* = 3 mice (unpaired Students *t*‐test).

Repeated daily injections of MuSK MG patient IgG into mice lead to slow progressive declines in endplate AChR density and endplate potential amplitude, culminating in failure of neuromuscular transmission after 12–15 days (Morsch et al. 2012). We used phosphospecific antibodies to examine the impact of anti‐MuSK IgG at the beginning of the anti‐MuSK injection series. Mice were given a single i.p. injection of MuSK MG IgG (batch AM4.5; 35 mg) or non‐myasthenic control IgG and were killed for analysis 24 h later (Fig. 1). Endplates of the control mice showed intense staining for phosphorylated Src‐Y418 (pSrc; Fig. 2A–C), AChR *β*‐subunit‐Y390 (pAChR; Fig. 2G–I), and total AChR (Alexa647‐*α*‐BGT binding), consistent with previous findings (Ghazanfari et al. 2014). After the single injection of MuSK MG IgG, endplates revealed no measurable reduction in total AChR labeling intensity compared to control mice (Fig. 2D,J&M), consistent with previous findings (Morsch et al. 2012). In contrast, immunolabeling for pSrc and pAChR were both significantly reduced (Fig. 2E,K&M). Thus, in the mouse passive IgG transfer model of MuSK MG, anti‐MuSK IgG caused rapid reductions in pSrc and pAChR at the endplate, prior to any measurable decline of endplate AChR density.

### Synaptic targeting of MuSK‐EGFP in healthy mice

**Figure 3:**
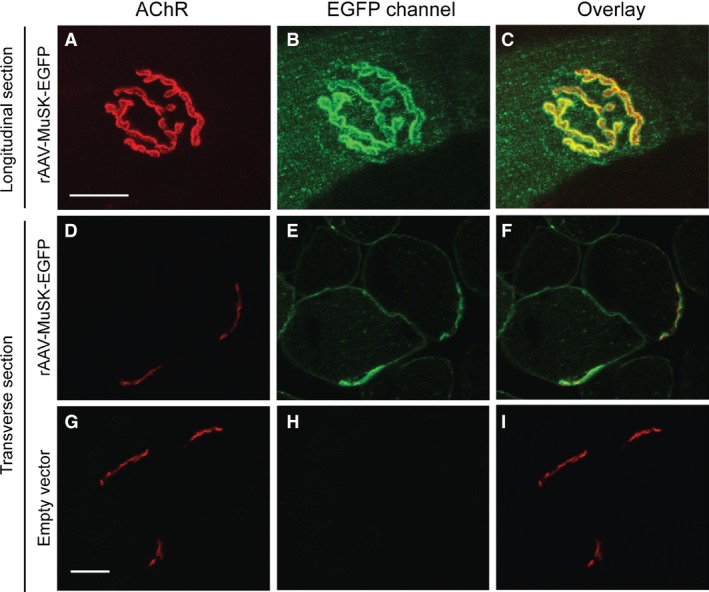
Subcellular targeting of MuSK‐EGFP in healthy muscle fibers. The mouse TA muscle was injected with rAAV encoding MuSK‐EGFP (2 × 10^9^ vg). Three weeks later, mice were killed and the muscle sectioned. (A) Longitudinal section Z‐projection image showing an endplate viewed enface, labeled for AChR (Alexa555‐*α*‐BGT). (B) MuSK‐EGFP fluorescence at the same endplate (C). Overlay of the two fluorescence channels. (D) Transverse section of the TA muscle. AChR labeling reveals two endplates on adjoining muscle fibers. In transverse section, endplates often appear crescent shaped. (E) MuSK‐EGFP fluorescence in the same microscope field. (F) Overlay of the two fluorescence channels. (G–I) Transverse section of the contralateral control muscle (injected with empty rAAV vector). AChR‐labeled endplates show no fluorescence in the EGFP channel. Scale bars = 20 *μ*m.

**Figure 4:**
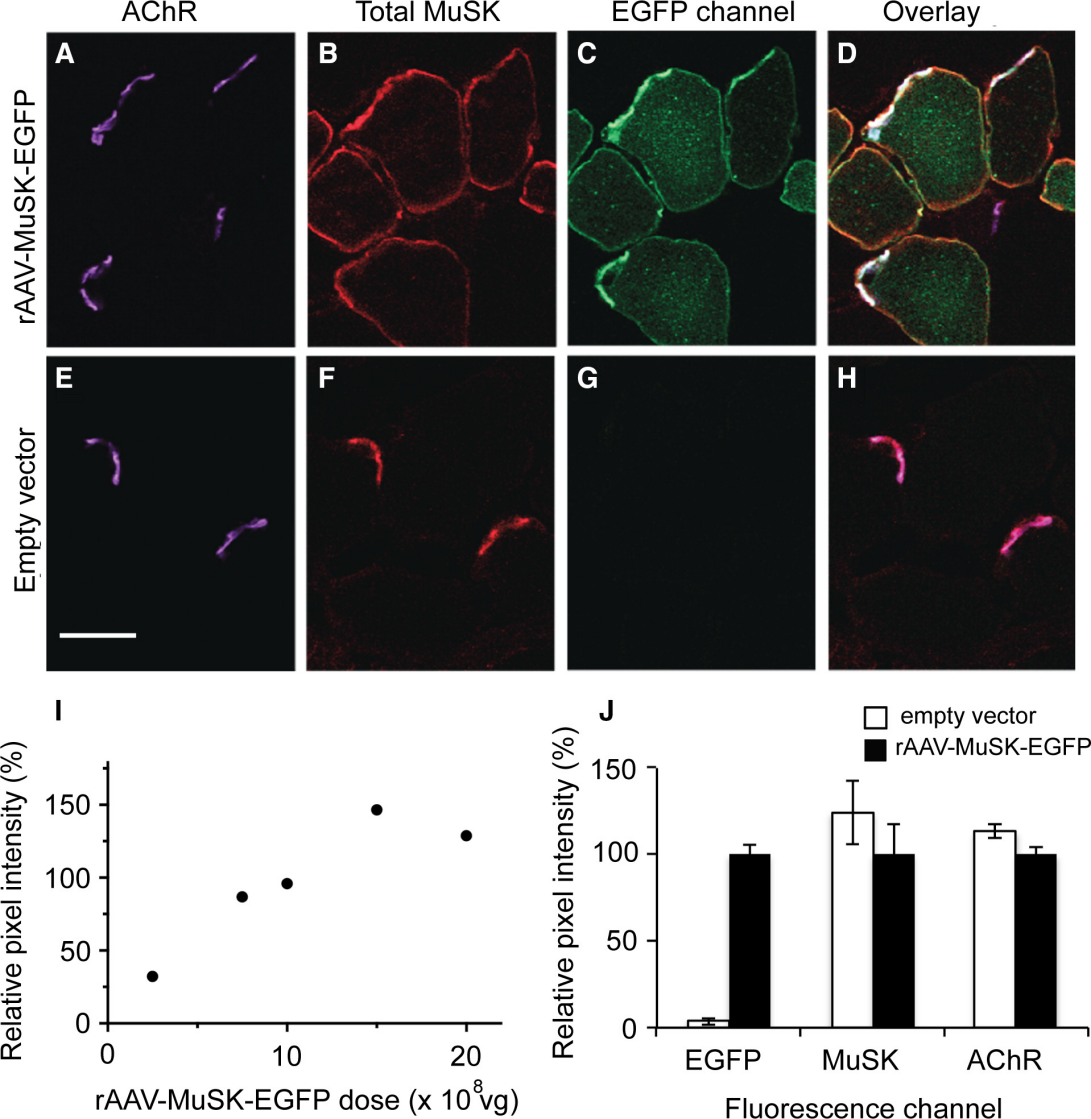
Forced expression of MuSK‐EGFP in healthy muscles does not increase the endplate density of MuSK or AChR. (A) Transverse section through endplates labeled for AChR (Alexa647‐*α*‐BGT) 3 weeks after i.m. injection of rAAV‐MuSK‐EGFP. (B) The same microscope field showing immunofluorescence to reveal total MuSK. (C) EGFP fluorescence channel. Note the mosaic expression: most, but not all, muscle fibers expressed MuSK‐EGFP. (D) Overlay of the three fluorescence channels. Endplate co‐localization is indicated by white pixels. (E–H) Endplates from the contralateral control muscle (injected with empty rAAV vector) show endogenous MuSK at endplates. The brightness of the digital images was increased for reproduction. The same percentage increase was applied to control and experimental panels. Scale bar = 20 *μ*m. (I) Effect of rAAV‐MuSK‐EGFP dose (number of vg injected) upon the relative intensity of MuSK‐EGFP fluorescence at the endplate. (J) Effects of MuSK‐EGFP upon the endplate pixel intensity for: MuSK‐EGFP fluorescence (EGFP), total MuSK immunofluorescence (MuSK), and AChR labeling (2 × 10^9^ vg injected). Filled columns show the fluorescence intensity at MuSK‐EGFP‐positive endplates relative to endplates in contralateral muscles injected with empty rAAV vector (open columns; mean ± SEM for *n* = 3 mice).

Recombinant adeno‐associated viral vector (rAAV) was used to increase the synthesis of MuSK within the muscle fibers. Three weeks after i.m. injection of rAAV into the TA muscle of naïve mice, MuSK‐EGFP fluorescence was evident in the sarcolemma, and was concentrated at the motor endplate (Fig. 3). In a pilot dose–response experiment, the intensity of MuSK‐EGFP fluorescence at the endplate reached a plateau with injection of 2 × 10^9^ vg of rAAV (Fig. 4I). Some MuSK‐EGFP‐expressing fibers also revealed punctate intracellular EGFP fluorescence with a mean diameter of 1 *μ*m (Figure S3). Immunolabeling for MuSK is normally restricted to the motor endplate (Fig. 4E–H; Cole et al. 2010; Ghazanfari et al. 2014). Muscles expressing MuSK‐EGFP revealed additional MuSK immunolabeling extending right around the circumference of the muscle fiber, consistent with supraphysiological expression levels (Fig. 4B). Despite the overall increase in MuSK abundance, the average intensity of immunolabeling for MuSK at the endplate (total MuSK) did not increase, compared to endplates in contralateral muscles that were injected with empty vector (Fig. 4J “MuSK”). Thus, in healthy muscles, the endplate density of MuSK appears to have a ceiling.

### Effects of acute anti‐MuSK challenge upon synaptic targeting of MuSK‐EGFP

**Figure 5:**
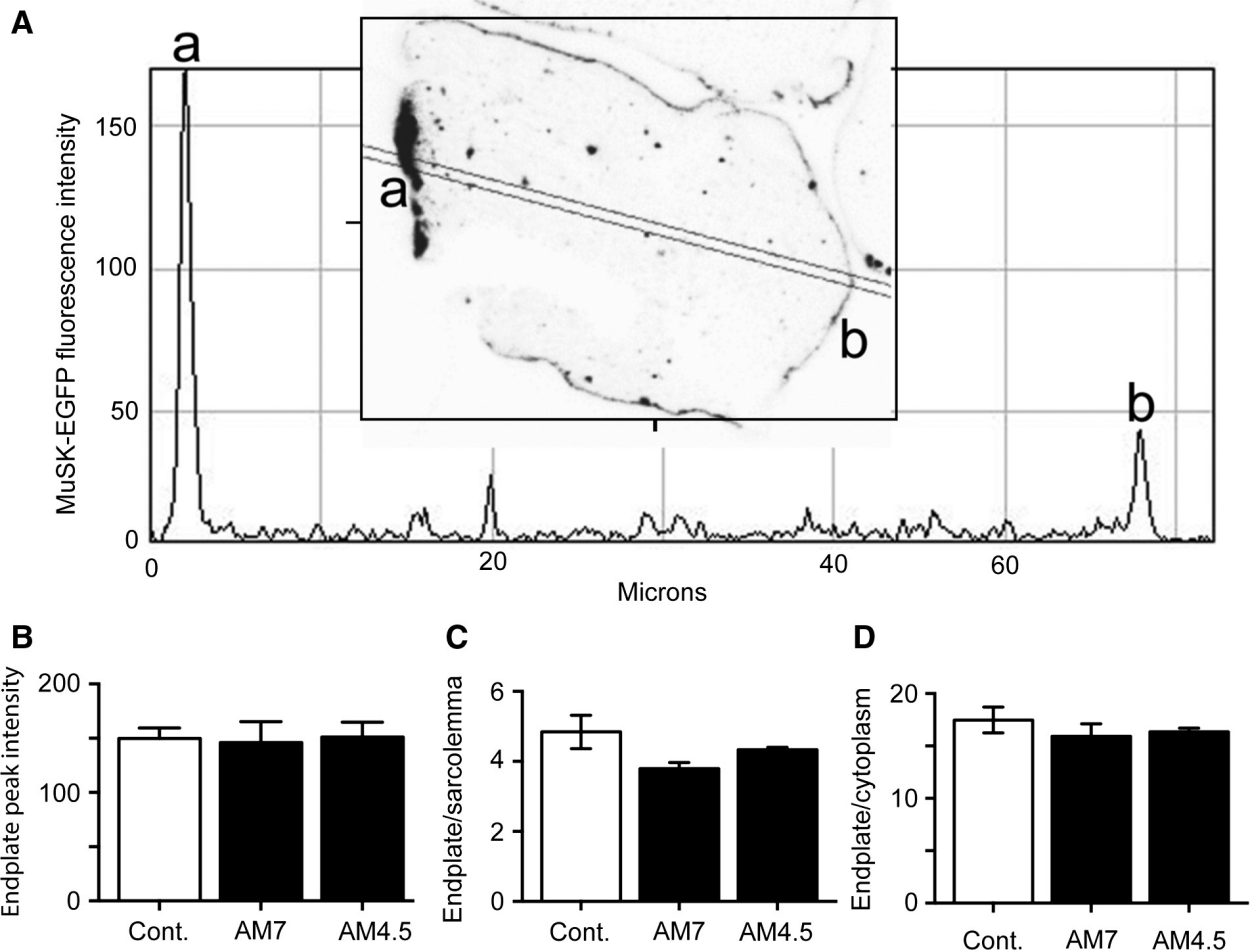
A single injection of MuSK MG IgG does not alter the synaptic localization of MuSK‐EGFP. Mice expressing MuSK‐EGFP in their TA muscles were challenged with a single i.p. injection of MuSK MG patient IgG (25 mg AM7.2 or 35 mg AM4.5) or control IgG (35 mg). Twenty‐four hours later mice were killed for analysis. (A) A sample MuSK‐EGFP fluorescence intensity profile. The inset shows a transverse optical cross section through the endplate portion of a muscle fiber. The digital image has been inverted, so that MuSK‐EGFP fluorescence appears dark on a light background. The intensity profile represents the twin line in the inset. Peaks correspond to the endplate (a) and the extrasynaptic sarcolemma (b). Cytoplasmic EGFP intensity was averaged between the two peaks. (B) Endplate peak intensities for mice injected with AM7 IgG, AM4.5 IgG and non‐myasthenic control IgG (Cont.). Average fluorescence pixel intensity is shown in arbitrary units. (C) Endplate peak intensity relative to the extrasynaptic sarcolemma peak intensity. (D) Endplate peak intensity relative to average cytoplasmic intensity. None of the small differences in mean values were statistically significant (n.s.; *P* > 0.05). Bars represent the mean ± SEM for *n* = 3–4 mice (one‐way ANOVA with Bonferroni's multiple comparisons post‐test).

MuSK‐EGFP was used first to probe the acute effects of anti‐MuSK IgG upon synaptic targeting. Mice expressing MuSK‐EGFP in their TA muscle were given a single i.p. injection of IgG from a MuSK MG patient (patient batch AM7 or AM 4.5) or control IgG and were killed for analysis 24 h later. In mice that received control IgG, MuSK‐EGFP fluorescence was approximately fourfold more concentrated at the endplate compared to the extrasynaptic sarcolemma (Fig. 5A&C open bars) and was approximately 17 times more concentrated at the endplate compared to the cytoplasm (Fig. 5D). These values were comparable to mice that had not received any IgG (Figure S2). The single injection of MuSK MG IgG (AM4.5 or AM7) did not alter the mean intensity of MuSK‐EGFP fluorescence at the endplate (Fig. 5B compare filled bars with open bar), nor the degree to which MuSK‐EGFP remained concentrated in the postsynaptic membrane (Fig. 5C&D). Thus, 24 h exposure to MuSK MG IgG did not displace MuSK‐EGFP from the endplate.

### Elevated MuSK synthesis ameliorates anti‐MuSK‐induced synaptic impairment

**Figure 6:**
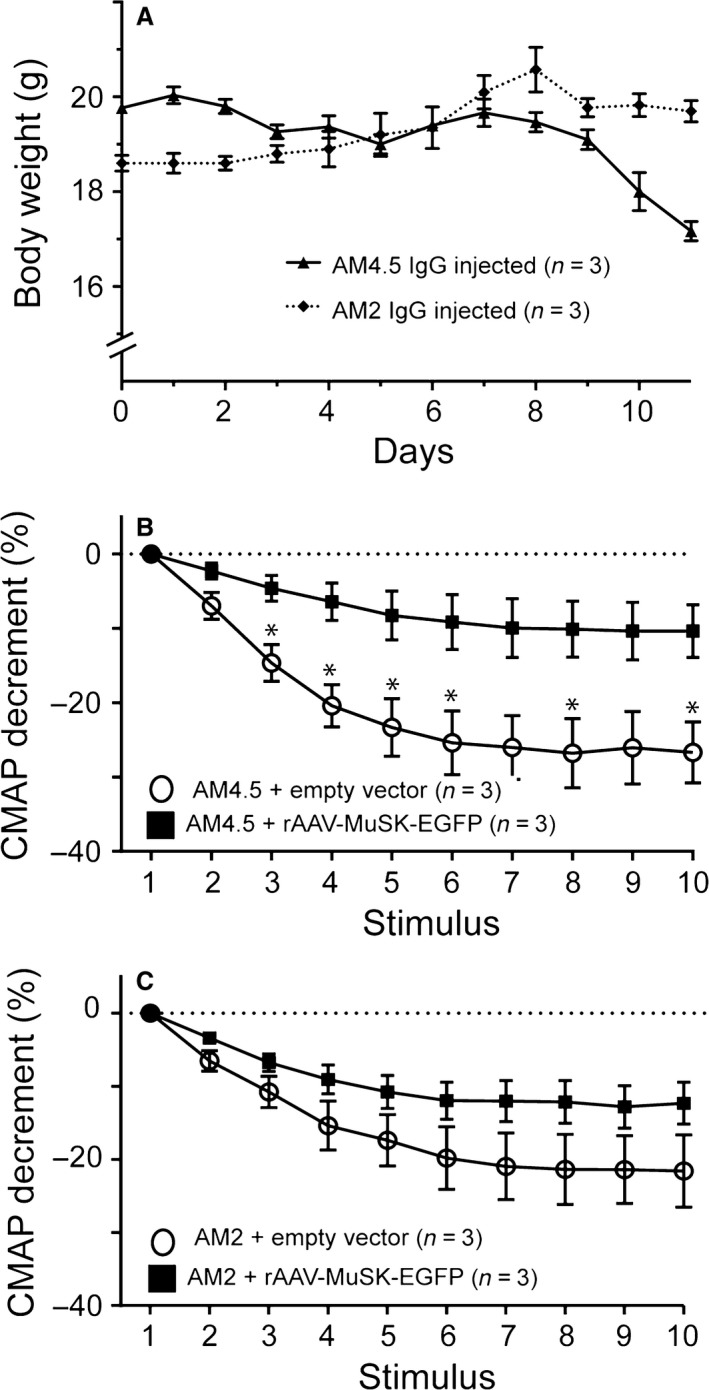
Effects of forced MuSK‐EGFP expression upon neuromuscular synapse function in our mouse model of MuSK MG. All mice received a unilateral injection of rAAV‐MuSK‐EGFP in their right TA muscle. The contralateral TA muscle received empty rAAV vector. Two weeks later, mice were challenged with a series of 11 daily i.p. injections of MuSK‐MG IgG. (A) Body weights of mice during the AM4.5 IgG and AM2 IgG injection series (35 and 45 mg/day, respectively; mean ± SEM for *n* = 3 mice). (B) Decrement in the CMAP amplitude during repetitive stimulation of the sciatic nerve (3 impulses per second) recorded from the TA muscles of mice after 11 daily injections of AM4.5 IgG. The MuSK‐EGFP‐expressing right TA muscle (filled symbols) displayed less decrement than contralateral, empty vector, muscles (open circles; mean ± SEM for *n* = 3 mice; *P < 0.05 unpaired *t*‐tests) (C) Comparable CMAP results for mice injected with AM2 IgG, except that differences in CMAP amplitude did not reach statistical significance.

While a single injection of MuSK MG IgG did not displace MuSK‐EGFP from the endplate, repeated daily injections of IgG from MuSK MG patients have been shown to reduce endplate levels of both MuSK and AChR (Cole et al. 2010; Ghazanfari et al. 2014). We tested whether MuSK‐EGFP could ameliorate this loss of MuSK and AChR. The right TA muscle was administered rAAV‐MuSK‐EGFP while the contralateral muscle received empty rAAV vector. Mice were subsequently challenged with 11 daily injections of MuSK MG IgG (Fig. 1) so as to produce a robust reduction in endplate AChRs without the whole‐body weakness that occurs after a longer injection series (Morsch et al. 2012). Accordingly mice injected with AM4.5 IgG (35 mg/day) exhibited only a modest weight loss by day 11 while mice injected with AM2 (45 mg/day) did not show any noticeable change in body weight (Fig. 6A). None of the mice displayed impaired posture or mobility according to standard criteria (Phillips et al. 2015). Nevertheless, compound muscle action potential (CMAP) recordings revealed evidence of “subclinical” impairment of neuromuscular transmission, as expected (Morsch et al. 2012). Recordings from the left TA muscles (empty vector) revealed a marked decrement in the CMAP amplitude during repetitive nerve stimulation (3 impulses/sec; Fig. 6B open circles). In contrast, right TA muscles (MuSK‐EGFP‐expressing) from the same mice displayed significantly less decrement (Fig. 6B filled squares). Recordings of CMAPs from mice injected with AM2 IgG (which showed no weight loss) revealed qualitatively similar results, but the trend toward amelioration of the CMAP decrement in the MuSK‐EGFP‐expressing muscles did not reach statistical significance (Fig. 6C).

**Figure 7:**
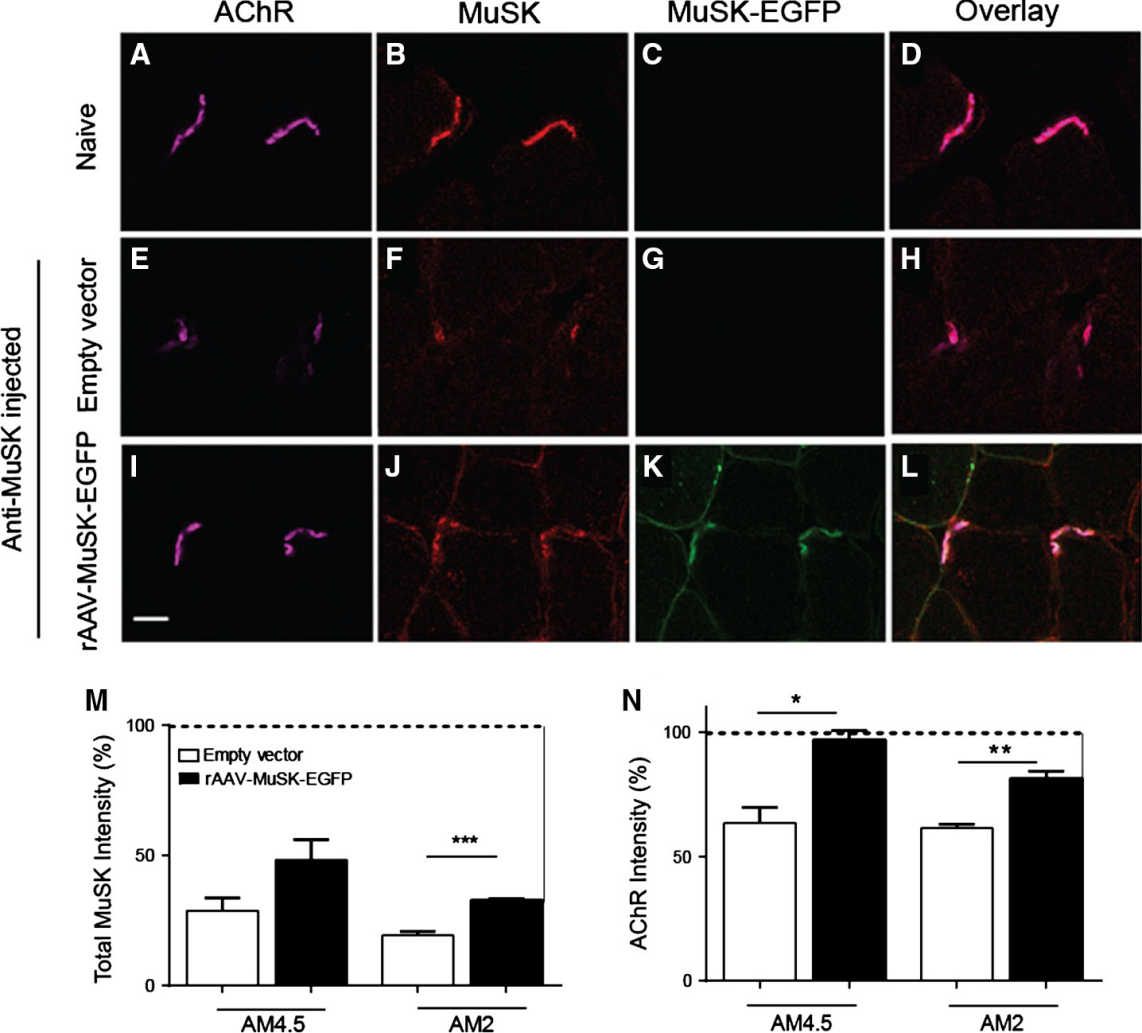
Increased expression of MuSK‐EGFP ameliorates the loss of endplate AChR in our mouse model of MuSK MG. Transverse sections of TA muscles from the mice described in Figure 6 were examined. (A) Two endplates from a naïve mouse illustrate the normal bright labeling of AChR with Alexa647‐*α*‐BGT. (B) The same microscope field showing immunofluorescence for total MuSK. (C) The EGFP fluorescence channel. (D) Overlay of the three fluorescence channels. (E‐H) Two representative endplates from the left TA muscle (empty vector) after 11 daily injections of AM4.5 IgG (35 mg/day). Only weak AChR and MuSK labeling remains. (I–L) Representative endplates from the right TA muscle (rAAV‐MuSK‐EGFP) of the same mouse. Endplates expressing MuSK‐EGFP retain relatively bright labeling for AChR and total MuSK. Scale bar = 20 *μ*m. (M) Effect of MuSK‐EGFP upon the intensity of MuSK immunofluorescence (total MuSK) at endplates after 11 daily injections of AM4.5 (35 mg/day) or AM2 (45 mg/day). Black bars represent the MuSK‐EGFP‐expressing right TA muscle. Open bars represent contralateral muscles (injected with empty vector). (N) Effect of MuSK‐EGFP upon the intensity of endplate AChR labeling in the same muscles. For panels M and N, average fluorescence intensities for individual mice were normalized to the mean for healthy endplates of naïve mice (horizontal dashed line). Bars represent mean SEM for *n* = 3 mice (*P < 0.05, **P < 0.01; ***P < 0.001; unpaired Student's *t*‐test).

**Figure 8:**
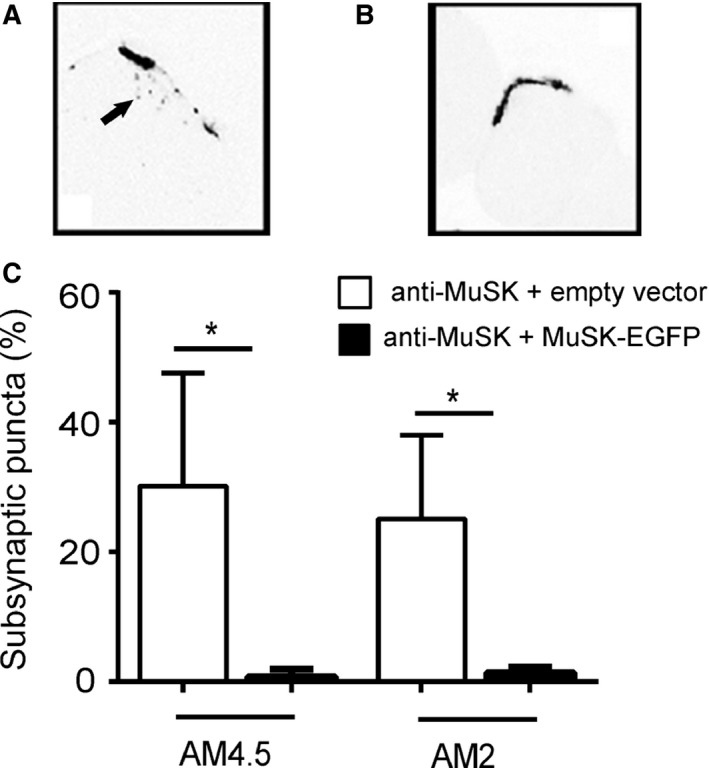
Increased expression of MuSK prevents the appearance of subsynaptic puncta of AChR in our mouse model of MuSK MG. (A) A transverse section through an endplate from the left TA muscle (empty rAAV) after 11 daily injections of AM4.5 IgG (35 mg/day). AChRs are labeled with Alexa647‐*α*‐BGT. Tiny puncta of AChR labeling (arrow) are evident in the cytoplasm beneath the endplate. The digital image has been inverted, so that MuSK‐EGFP fluorescence appears dark on a light background. (B) A MuSK‐EGFP‐positive endplate from the same mouse (EGFP channel not shown). No punctate AChR labeling is evident beneath the endplate. Scale bar is 20 *μ*m. (C) Percentage of endplates displaying subsynaptic puncta AChR labeling after 11 daily injections of either AM4.5 (35 mg/day) or AM2 (45 mg/day). Open bars show results for the left TA muscle (empty vector) compared with MuSK‐EGFP‐expressing right TA muscle (black bars; mean ± SEM for *n* = 3 mice, *P < 0.05, paired Student's *t*‐test).

Endplates expressing MuSK‐EGFP retained more of the normal complement of MuSK immunolabeling after the anti‐MuSK injection series than for contralateral muscles that had been injected with empty vector (Fig. 7F,J&M). Importantly, endplates that expressed MuSK‐EGFP also retained more intense AChR labeling compared to contralateral (empty vector) muscles (Fig. 7E,I&N). The loss of endplate AChR after repeated daily injections of MuSK MG IgG is often accompanied by the appearance of tiny puncta of AChR labeling in the cytoplasm beneath the endplate (Ghazanfari et al. 2014). Subsynaptic puncta of AChR were again evident in the left TA muscles (empty vector) after 11 daily injections of MuSK MG IgG (Fig. 8A), but were absent from endplates expressing MuSK‐EGFP (Fig. 8B&C). Together, these results suggest that increased expression of MuSK can inhibit the loss of AChR from the endplate that normally results from repeated injections of MuSK MG IgG.

### Rapsyn‐EGFP did not protect against anti‐MuSK‐induced AChR loss

**Figure 9:**
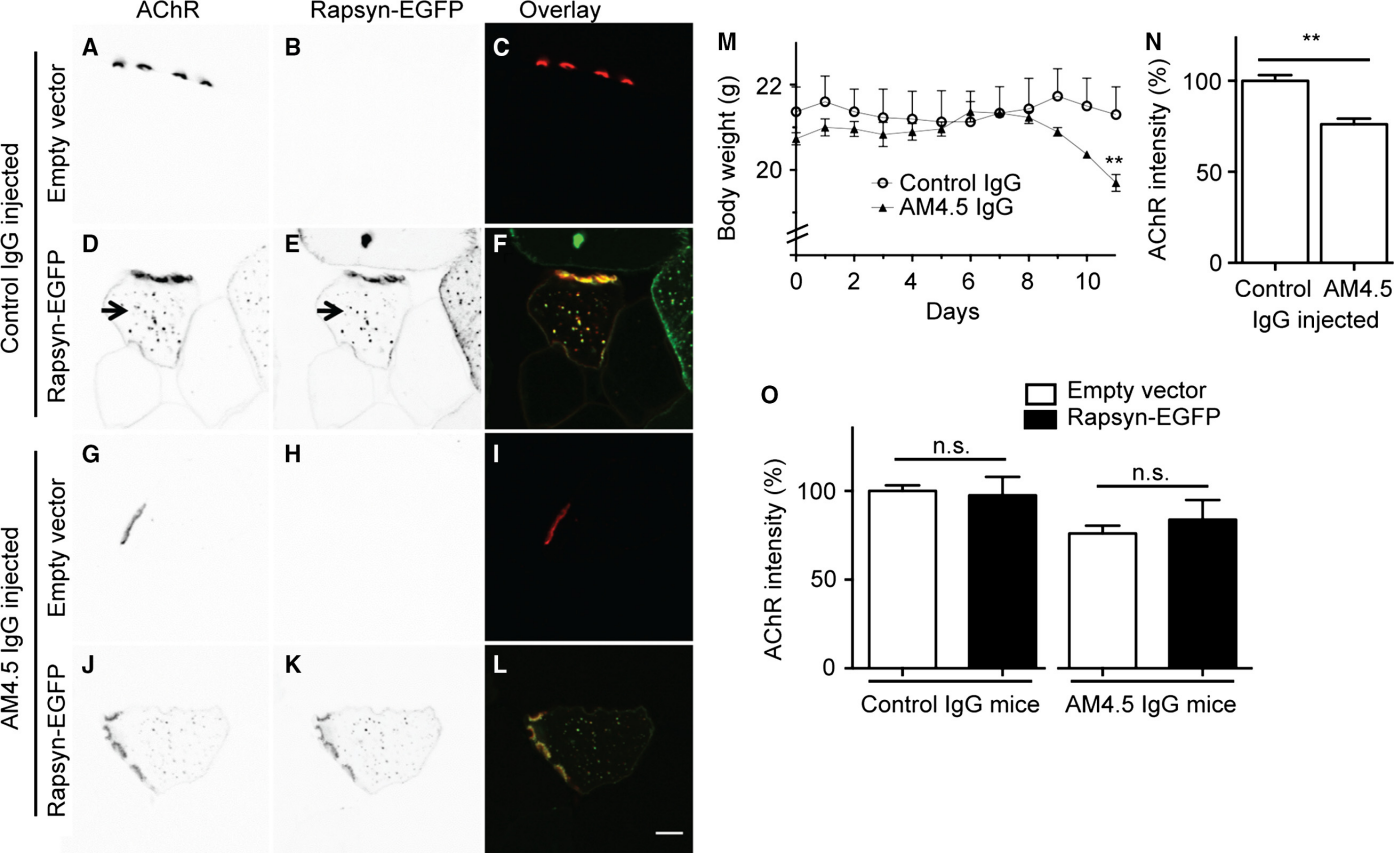
Distribution of rapsyn‐EGFP and AChR at endplates and the effects of MuSK autoantibodies. Mice injected with rAAV‐rapsyn‐EGFP in their right TA muscle and empty vector in their left TA muscle were subsequently challenged with 11 daily i.p. injections of non‐myasthenic control IgG or AM4.5 IgG (35 mg/day). (A–C) Transverse optical section through the left TA muscle of a mouse injected with control IgG shows bright AChR labeling of an endplate (Alexa647‐*α*‐BGT). (D–F) A section through the right TA muscle of the same mouse shows bright rapsyn‐EGFP and AChR labeling of the endplate. Rapsyn‐EGFP and AChR labeling are also co‐localized in puncta beneath the endplate (arrow). (G–I) Section through the left TA muscle of a mouse injected with AM4.5 IgG shows relatively dim AChR labeling of a myasthenic endplate. (J–L) A section through the right TA muscle of the same mouse shows relatively dim endplate AChR labeling despite the presence of rapsyn‐EGFP. Scale bar is 10 *μ*m. (M) Body weight of mice during the IgG injection series (mean ± SEM,* n* = 3 mice). Mice receiving AM4.5 IgG showed a significant loss of weight on the last day of the injection series compared to mice injected with control IgG (**P < 0.01; unpaired *t*‐test). (N) The intensity of endplate AChR labeling was reduced at the end of the AM4.5 IgG injection series, compared to mice injected with control IgG. Open bars show mean Alexa647‐*α*‐BGT fluorescence intensity ± SEM for endplates from empty vector‐injected left TA muscles (unpaired *t*‐test, *n* = 3 mice). (O) Paired comparison of endplate AChR labeling intensity at rapsyn‐EGFP‐expressing endplates (black bars) versus contralateral, empty vector, control muscles (open bars; n.s. = *P* > 0.05; paired *t*‐test).

An additional group of mice received an i.m. injection of rAAV encoding rapsyn‐EGFP (2 × 10^9^ vg) into their right TA muscle (with empty vector into the contralateral muscle). The mice were subsequently challenged with 11 daily injections of MuSK MG IgG (AM4.5; 35 mg/day) or non‐myasthenic control IgG, as above. In mice injected with control IgG, rapsyn‐EGFP targeted strongly to the endplate and to small cytoplasmic puncta (Fig. 9D–F). This is consistent with the subcellular distribution of rapsyn‐EGFP found in a previous study (Gervásio and Phillips 2005). Many of the rapsyn‐EGFP puncta that occurred beneath the endplate in these healthy mice also labeled positive for AChR (Fig. 9D–E arrow). Mice that received 11 daily injections of AM4.5 IgG showed a small reduction in body weight (Fig. 9M), and a significant reduction in endplate AChR density (Fig. 9G&N), compared to mice injected with control IgG. Rapsyn‐EGFP expression did not rescue endplate AChR labeling intensity compared to contralateral (empty vector‐injected) muscles (Fig. 9J&O). Thus, forced expression of rapsyn‐EGFP did not significantly protect against anti‐MuSK‐induced loss of AChR from the endplate.

## Discussion

This study supports the idea that MuSK autoantibodies cause the loss of postsynaptic AChR by inhibiting the synapse maintenance function of MuSK. MuSK autoantibodies from MG patients are predominantly of the IgG4 subtype and studies in rodent models indicate that their harmful effects are not dependent upon classical complement‐mediated immunopathology (McConville et al. 2004; Klooster et al. 2012; Mori et al. 2012; Phillips et al. 2015). In vitro assays show that MuSK autoantibodies can inhibit assembly of the neural agrin‐LRP4‐MuSK complex and thereby might prevent the “physiological” activation of MuSK (Huijbers et al. 2013; Koneczny et al. 2013; Otsuka et al. 2015). These findings suggest that interruption of agrin‐MuSK signaling activity might be sufficient to cause NMJ disassembly. Our in vivo findings now confirm that MuSK autoantibodies rapidly suppress tyrosine phosphorylation of targets of the MuSK pathway within the postsynaptic membrane. However, repeated passive transfer of MuSK MG IgG into mice eventually leads to depletion of endplate MuSK immunolabeling and AChR density (Cole et al. 2010; Kawakami et al. 2011; Ghazanfari et al. 2014; Otsuka et al. 2015). Conversely, a forced increase in MuSK synthesis ameliorated these effects of MuSK autoantibodies in our mouse passive transfer model of MuSK MG. Together, these findings suggest that the autoantibodies cause synapse failure by reducing the activity of MuSK and subsequently MuSK expression at the motor endplate.

Immunolabeling for pSrc and pAChR provides an indication of potential changes in the tyrosine kinase activity of MuSK within the postsynaptic membrane. Endplates of healthy mice displayed intense immunolabeling for both pSrc and pAChR, but the intensity of both was reduced after 14 daily injections of MuSK MG IgG (Ghazanfari et al. 2014). We questioned whether this might have been secondary to other pathophysiological changes. Here, we show that prominent reductions in pSrc and pAChR immunolabeling of endplates were evident within 24 h of the first injection of MuSK MG IgG, prior to measurable displacement of MuSK and loss of AChR. This timing is consistent with the notion that reduced MuSK kinase activity is the primary cause of the subsequent loss of endplate AChR. There is reason to suspect that a reduction in AChR phosphorylation might contribute to the subsequent loss of endplate AChRs. When neural agrin is added to cultured muscle cells, it activates MuSK leading to phosphorylation of Src and the AChR. AChR phosphorylation recruits rapsyn and helps link AChR to the cortical cytoskeleton and to protein kinase A (Borges and Ferns 2001; Borges et al. 2008; Choi et al. 2012). Forced expression of the intracellular protein, DOK7 (which enhances the activation of MuSK), increased the phosphorylation of AChR and the size of endplate AChR clusters (Arimura et al. 2014). Conversely, knock‐in mutations that precluded tyrosine phosphorylation of the AChR *β*‐subunit caused a small, but significant reduction in the density of AChR at the adult motor endplate (−10%; Friese et al. 2007). Thus, anti‐MuSK‐induced suppression of MuSK kinase activation could plausibly explain the loss of endplate MuSK and AChR density.

Forced expression of MuSK protected endplates from AChR loss despite circulating autoantibodies that are thought to block the activation of MuSK. This paradox might be explained by earlier studies conducted in healthy mice. Forced expression of MuSK in the extrasynaptic portion of the muscle fibre induced the formation of endplate‐like AChR clusters, even in experiments that precluded the interaction of MuSK with LRP4 and agrin (Sander et al. 2001; Kim and Burden 2008). Thus, even without activation by agrin and LRP4, the intrinsic kinase activity of MuSK can be sufficient to organize postsynaptic AChRs into clusters, provided the density of MuSK in the membrane is sufficiently high. Forced expression of MuSK in our healthy mice did not significantly increase the endplate density of MuSK. In mice receiving repeated injections of anti‐MuSK, the early reduction in endplate protein phosphorylation (Fig. 2), was followed by reductions in the endplate density of MuSK of as much as 75% (Fig. 7; Cole et al. 2010; Kawakami et al. 2011; Ghazanfari et al. 2014). Forced expression of MuSK‐EGFP ameliorated this autoantibody‐induced loss of MuSK from the endplate, by maintaining about half the healthy endplate density of MuSK (Fig. 7M&N). Thus, if the activating influence of agrin‐LRP4 is interrupted by autoantibodies, retention of postsynaptic AChRs may depend upon the endplate maintaining a certain minimum density of MuSK.

The effects of rapsyn‐EGFP appeared to differ from those of MuSK‐EGFP. Consistent with previous findings, rapsyn‐EGFP targeted precisely to the postsynaptic AChR cluster. Rapsyn also targets to trans‐Golgi elements where it normally assembles with newly synthesized AChR (Marchand et al. 2002; Gervásio and Phillips 2005). Consistent with this, AChR labeling was co‐localized with some of the cytoplasmic puncta of rapsyn‐EGFP (Fig. 9D–F). Forced expression of rapsyn was found to protect endplate AChRs in a rat model of MG that involves passive transfer of monoclonal anti‐AChR (Losen et al. 2005). Rapsyn‐EGFP had no significant protective influence in our passive transfer model of MuSK MG (Fig. 9). Post hoc analysis the MuSK‐EGFP results (Fig. 7N) suggests an effect size of 1.65 and statistical power of 0.52 for our experiments (*n* = 3 mice). Hence, the lack of effect of rapsyn‐EGFP in Figure 9 might simply represent type II error. However, MuSK activation has two established effects on rapsyn. Firstly, it slows the proteolysis of rapsyn, thereby increasing the cytoplasmic rapsyn pool (Brockhausen et al. 2008; Luo et al. 2008). Secondly, phosphorylation of the AChR *β*‐subunit recruits extra rapsyn from the cytoplasmic pool to bind to the AChR (Borges et al. 2008). A large pool of rapsyn‐EGFP may be of little benefit when tyrosine phosphorylation of the AChR is impaired.

The present results suggest that human MuSK autoantibodies initiate a cascade of pathophysiological changes at the endplate. Inhibition of MuSK activation causes a marked reduction in the intensity of endplate immunolabeling for pSrc and pAChR, consistent with the in vitro finding that MuSK MG IgG4 blocks the activation of the MuSK complex by neural agrin (Huijbers et al. 2013; Koneczny et al. 2013; Otsuka et al. 2015). This seems to be followed by a delayed displacement of MuSK and AChR from the endplate. Forced expression of MuSK ameliorated the autoantibody‐induced loss of both MuSK and AChR from the endplate. Further detailed study of the effects of MuSK MG autoantibodies should help deepen our understanding of the mechanisms of postsynaptic differentiation and may reveal new therapeutic strategies for MuSK MG.

## Conflict of Interest

None declared.

## Supporting information




**Figure S1.** Percentage of MuSK‐EGFP‐positive endplates increased with the dose of rAAV injected. The tibialis anterior muscle was injected with the indicated number of viral genomes (vg) of rAAV that encoded MuSK‐EGFP. The percentage of AChR‐stained endplates that were positive for MuSK‐EGFP fluorescence was counted from sampled images.
**Figure S2.** Dosage of rAAV‐MuSK‐EGFP did not alter the relative distribution of MuSK‐EGFP at the motor endplate. Tibialis anterior muscles were injected with the indicated range of dosages of rAAV‐MuSK‐EGFP (2.5–20 × 10^8^ viral genomes per muscle). Three weeks later the mice were killed and muscles fixed for confocal imaging. (A) Peaks of MuSK‐EGFP fluorescence intensity corresponding to the postsynaptic and extrasynaptic portions of the muscle fibre peripheral membrane were compared by a line drawn perpendicular to the motor endplate. (B) Ratio of peak endplate MuSK‐EGFP fluorescence to peak fluorescence in the extrasynaptic sarcolemma. (C) Ratio of peak endplate MuSK‐EGFP fluorescence to the average fluorescence intensity within the cytoplasm.
**Figure S3.** Intracellular puncta of MuSK‐EGFP. (A) Transverse section through a fibre expressing high levels of MuSK‐EGFP at the endplate and in multiple cytoplasmic puncta. Note the lower levels of MuSK‐EGFP expression, and fewer punta evident, in neighbouring fibres. Such puncta, and sarcolemmal MuSK‐EGFP fluorescence extended beyond the endplate portion of many fibres. This particular muscle was injected with 1 × 10^9^ vg of rAAV‐MuSK‐EGFP. (B) Frequency histogram showing the Gaussian distribution for the diameter of EGFP fluorescent cytoplasmic puncta (1.38 ± 0.36 *μ*m; *n *= 229 puncta).
