# Supplementary material for: Forced expression of muscle specific kinase slows postsynaptic acetylcholine receptor loss in a mouse model of MuSK myasthenia gravis
Source: Physiol Rep. 2015 Dec 23;3(12):e12658. doi: 10.14814/phy2.12658 (PMC4760443; doi:10.14814/phy2.12658)
Supplement: Supplementary file 1 — Figure S1. Percentage of MuSK‐EGFP‐positive endplates increased with the dose of rAAV injected. The tibialis anterior muscle was injected with the indicated number of viral genomes (vg) of rAAV that encoded MuSK‐EGFP. The percentage of AChR‐stained endplates that were positive for MuSK‐EGFP fluorescence was counted from sampled images. Figure S2. Dosage of rAAV‐MuSK‐EGFP did not alter the relative distribution of MuSK‐EGFP at the motor endplate. Tibialis anterior muscles were injected with the indicated range of dosages of rAAV‐MuSK‐EGFP (2.5–20 × 108 viral genomes per muscle). Three weeks later the mice were killed and muscles fixed for confocal imaging. (A) Peaks of MuSK‐EGFP fluorescence intensity corresponding to the postsynaptic and extrasynaptic portions of the muscle fibre peripheral membrane were compared by a line drawn perpendicular to the motor endplate. (B) Ratio of peak endplate MuSK‐EGFP fluorescence to peak fluorescence in the extrasynaptic sarcolemma. (C) Ratio of peak endplate MuSK‐EGFP fluorescence to the average fluorescence intensity within the cytoplasm. Figure S3. Intracellular puncta of MuSK‐EGFP. (A) Transverse section through a fibre expressing high levels of MuSK‐EGFP at the endplate and in multiple cytoplasmic puncta. Note the lower levels of MuSK‐EGFP expression, and fewer punta evident, in neighbouring fibres. Such puncta, and sarcolemmal MuSK‐EGFP fluorescence extended beyond the endplate portion of many fibres. This particular muscle was injected with 1 × 10^9^ vg of rAAV‐MuSK‐EGFP. (B) Frequency histogram showing the Gaussian distribution for the diameter of EGFP fluorescent cytoplasmic puncta (1.38 ± 0.36 μm; n = 229 puncta). [file PHY2-3-e12658-s001.pdf]

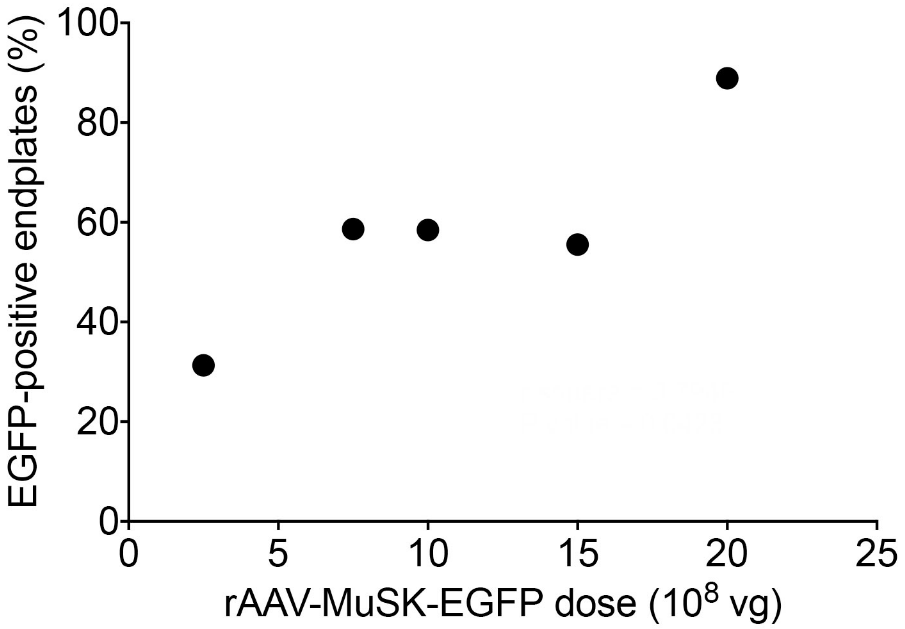

Supplementary Fig S1 Percentage of MuSK-EGFP-positive endplates increased with the dose of rAAV injected. The tibialis anterior muscle was injected with the indicated number of viral genomes (vg) of rAAV that encoded MuSK-EGFP. The percentage of AChR-stained endplates that were positive for MuSK-EGFP fluorescence was counted from sampled images.

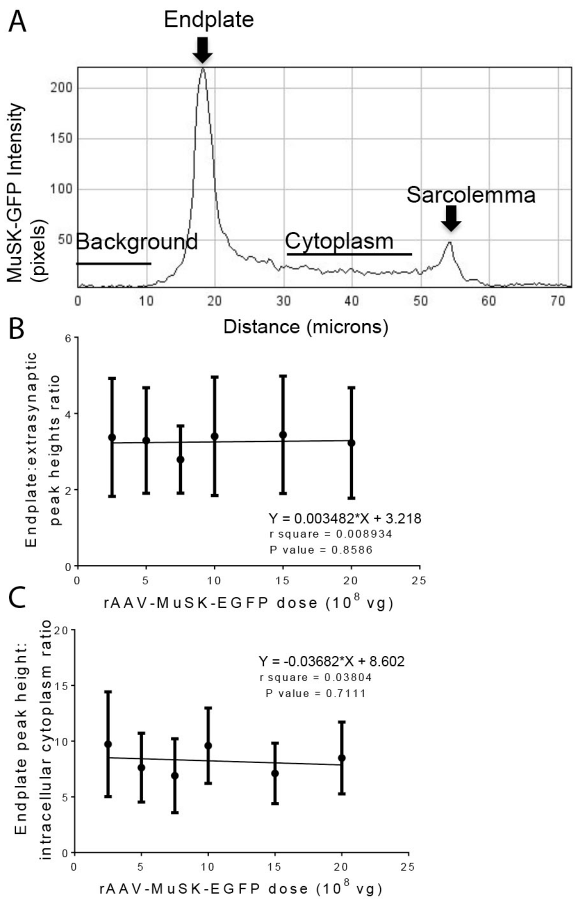

Supplementary Fig S2 Dosage of rAAV-MuSK-EGFP did not alter the relative distribution of MuSK-EGFP at the motor endplate. Tibialis anterior muscles were injected with the indicated range of dosages of rAAV-MuSK-EGFP (2.5 - 20 x 10^8 viral genomes per muscle). Three weeks later the mice were killed and muscles fixed for confocal imaging. (A) Peaks of MuSK-EGFP fluorescence intensity corresponding to the postsynaptic and extrasynaptic portions of the muscle fibre peripheral membrane were compared by a line drawn perpendicular to the motor endplate. (B) Ratio of peak endplate MuSK-EGFP fluorescence to peak fluorescence in the extrasynaptic sarcolemma. (C) Ratio of peak endplate MuSK-EGFP fluorescence to the average fluorescence intensity within the cytoplasm.

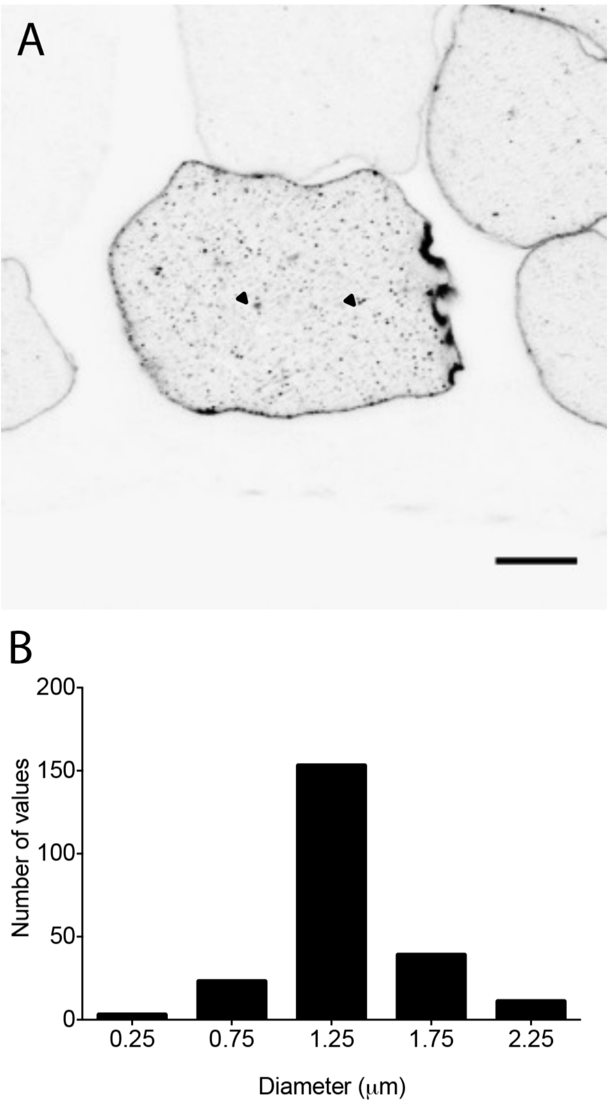

Supplementary Fig S3 Intracellular puncta of MuSK-EGFP. **(A)** Transverse section through a fibre expressing high levels of MuSK-EGFP at the endplate and in multiple cytoplasmic puncta. Note the lower levels of MuSK-EGFP expression, and fewer punta evident, in neighbouring fibres. Such puncta, and sarcolemmal MuSK-EGFP fluorescence extended beyond the endplate portion of many fibres. This particular muscle was injected with 1 x 10^9 v.g. of rAAV-MuSK-EGFP. **(B)** Frequency histogram showing the Gaussian distribution for the diameter of EGFP fluorescent cytoplasmic puncta (1.38 ± 0.36μm; n=229 puncta).
